# Integrative MeRIP-Seq and RNA-Seq Analyses Reveal Innate Immune and Infection-Related Transcriptomic Changes upon METTL3 Knockout

**DOI:** 10.3390/genes17070797

**Published:** 2026-07-13

**Authors:** Qian Tang, Yong Hu, Lin Zhu, Yue Liu, Jiaxin Zhang, Ziqian An, Qincai Dong, Cheng Cao

**Affiliations:** 1 School of Life Sciences and Medical Engineering, Anhui University, Hefei 230601, China; tangq6613@163.com; 2 National Key Laboratory of Advanced Biotechnology, Academy of Military Medical Sciences, Beijing 100071, China

**Keywords:** METTL3, mRNA methylation, anti-infection immune genes

## Abstract

**Background:** As a major regulator, methyltransferase-like 3 (METTL3) catalyzes N6-methyladenosine (m^6^A) modification in mRNA. The m^6^A modifications mediated by METTL3 influence RNA splicing, nucleocytoplasmic distribution, stability, and other functions, thereby playing a vital and indispensable role in genetic regulatory network. Although several studies have shown its critical role in mRNA fate, the global pattern of mRNA methylation alteration driven by METTL3 remain unclear. **Methods:** Here, a HEK293T cell line with METTL3 depletion was constructed, and RNA sequencing (RNA-seq) and methylated RNA Immunoprecipitation Sequencing (MeRIP-seq) were implemented. Additionally, quantitative Reverse Transcription PCR (qRT-PCR) technology was used to confirm some of the differentially expressed genes. **Result:** The mRNA methylation alteration landscape was clarified and the regions altered by m^6^A modification due to METTL3 deletion that was annotated and characterized, with 5763 hypomethylated/269 hypermethylated genes after METTL3 silence. Several methylation-related innate anti-infection immune genes, including *MYD88*, *RIG-1*, *CYLD* and *IRF9*, were exposed through comprehensive analysis to MeRIP-seq and RNA-seq data, and these genes were principally enriched in pathogen infection and innate immune response pathways such as *Shigellosis*, *Yersinia* infection, and the HIV-1 viral life cycle. **Conclusion:** Our study discovered that the METTL3 association with differentially expressed genes, suggested that METTL3 and the genes it regulates might serve as targets for defense against infection.

## 1. Introduction

Among eukaryotes mRNA, m^6^A is the most conservative and abundant modification accounting for more than 80% of base modification [1,2]. Its conserved recognition motif is DRACH (where D represents A, G, or U, R indicates G or A, and H denotes A, C, or U) [3,4], which frequently appear in the 3′UTR, upstream/downstream of the stop codon, and the exon regions [5,6,7]. m^6^A modification critically influences multiple metabolic processes, including mRNA stability, translation, splicing, and nucleocytoplasmic transport [8,9]. In addition to mRNA, m^6^A modification is widely found in other RNA molecules, including circular RNA (circRNA) [10,11] and long non-coding RNA (lncRNA) [12,13].

m^6^A modification is precisely orchestrated by a dynamic network comprising methyltransferases (writers), m^6^A-binding proteins (readers), and demethylases (erasers) [1,5,8,14]. As the core catalytic subunit of methyltransferase complex, METTL3 has been reported to involve in diverse biological processes, including embryonic development [15,16,17], stem cell self-renewal and fate determination [18,19,20,21,22,23], and tumorigenesis [2,14,24,25,26]. For example, knockdown of METTL3 reduces mRNA translation efficiency in female germ cells and markedly impedes oocyte maturation [27,28]. Emerging evidence has established a close connection between METTL3-mediated m^6^A modification and host innate immunity [10,29]. Collectively, METTL3 generally suppresses innate immune responses during viral infection [30,31]. For instance, in response to vesicular stomatitis virus infection, METTL3 catalyzes m^6^A methylation on viral RNAs, reduces viral double-stranded RNA formation, and thereby dampens *RIG-1* and MDA5-mediated antiviral signaling. Moreover, other study further demonstrated that METTL3 suppresses antiviral immune responses through two mechanisms: its catalytic motif impairs viral RNA recognition by *RIG-1* by means of m^6^A modification [32], whereas METTL3 also operates through non-catalytic motif, which recruits and stabilizes DDX3X by preventing its ubiquitination. Many studies report that deficiency of METTL3 function indeed leads to a severe reduction of m^6^A, impacting RNA stability, translation efficiency, and subcellular localization results in a transcriptional alteration of genes [23,26,33,34]. However, alteration in the global pattern of mRNA methylation mediated by METTL3 has not been exactly revealed. Although MeRIP-seq studies of METTL3 knockout have been reported in other cell types—such as colorectal cancer cells [26] and muscle stem cells [35]—our understanding of METTL3’s alteration in the global pattern of mRNA methylation in HEK293T cells, particularly in the context of innate immune genes, remains incomplete. Here, a CRISPR-mediated knockout of METTL3 cell line was established and MeRIP-seq was performed to detect the differentially methylated genes (DMGs) in m^6^A methylation-altered regions, and the significant differentially methylated mRNAs were annotated and characterized. In addition, RNA-seq was conducted to explore differentially expressed genes (DEGs). A set of innate anti-infection immune genes were obtained via combinatorial analysis, and were then analyzed for functional enrichment to identify the relevant pathways. Our study contributed to the increase in the general understanding of METTL3 to mRNA methylation alteration pattern, and underscores the latent significance of METTL3 in immune response against infection.

## 2. Materials and Methods

### 2.1. Cell Line Construction and Cultures

To construct the CRISPR-mediated METTL3 KO cell line, the sgRNA sequence 5′-GGAACTGCTGAAGCTGTGCTGGG-3′ targeting exon 2 of METTL3 gene was cloned into a lenti-Cas9 vector (lentiCRISPR v2, Addgene, Watertown, MA, USA, Plasmid#52961), and the vector, along with the pCMV-VSVG plasmid and the Gag-Pol plasmid, was transfected into HEK293T cells by lipofectamine 8000 (Beyotime Biotechnology, Shanghai, China, C0533) to produce lentivirus. After lentiviral transduction, cells were incubated for 6 h, followed by replacement with fresh medium. Post-infection 48 h, puromycin (Amresco, Solon, OH, USA, J593) was added to the culture medium, and the monoclonal METTL3 KO cell (clone #10) was obtained after selected with puromycin for 48–72 h, which was validated by Western blotting and DNA sequencing. Single-cell clones were obtained by limited dilution. And the negative control cell line was constructed with the sgRNA sequence 5′-GGATACTTCTTCGAACGTTT-3′. Both cell lines were established at Shanghai Obio Technology Corp., Ltd. and they were cultured in Dulbecco’s Modified Eagle Medium (DMEM) containing 10% fetal bovine serum, 100 mg/mL streptomycin, and 100 U/mL penicillin, at 37 °C with 5% CO_2_.

### 2.2. Reagents

DMEM (Gibco, Waltham, MA, USA, 11995065BT), fetal bovine serum (ExCell Bio, Shanghai, China, FSP500), penicillin (CSPC, Shijiazhuang, China, HB021634), streptomycin (North China Pharmaceutical Company. Ltd., Shijiazhuang, China, H20054140), PVDF membrane (Millipore, Burlington, MA, USA, IPVH00010), skim milk powder (BD *Difco*, Franklin Lakes, NJ, USA, 232100), anti-METTL3 (Cell Signaling Technology, Danvers, MA, USA, 86132S), RNeasy Mini Kit (Qiagen, Hilden, Germany, 74106), TBST (LABLEAD, Beijing, China, T720913), donkey anti-rabbit secondary antibody (Amersham, Buckinghamshire, UK, NA934V), Dual-Luciferase^®^ Reporter Assay System (Promega, Madison, WT, USA, E1910), anti-β-actin-HRP (Proteintech, Wuhan, China, HRP-60008), Luna^®^ One-Step RT-qPCR Kit (NEB, Ipswich, MA, USA, E3005L).

### 2.3. Plasmids

Ebola virus minigenome system plasmids, which include pCAGGS-L, pCAGGS-NP, pCAGGS-VP35, pCAGGS-VP30, pCAGGS-T7, p4cis-vRNA-Luc and pCAGGS-Tim1, were stored in our laboratory. The PGL3-promoter plasmid was purchased from UNIBIO (Chongqing, China). Plasmid transfection was carried out with Lipofectamine 3000 (Invitrogen, Carlsbad, CA, USA, L3000150) according to the instruction of the reagent manufacturer.

### 2.4. Quantitative Reverse Transcription PCR (qRT-PCR)

RNA was isolated with the RNeasy Mini Kit. Real-time quantitative PCR was conducted with the Luna^®^ Universal One-Step RT-qPCR Kit. The conditions included an initial denaturation at 95 °C for 2 min, then 40 cycles of amplification (15 s at 94 °C for denaturation, 1 min at 60 °C for annealing and extension). Relative expression levels of target genes were calculated using the 2^^(−ΔΔCT)^ method and normalized to the GAPDH. Experiment was repeated at least three times. The primer sequences are shown in Appendix A.

### 2.5. Western Blot

Protein lysates were prepared on ice in a buffer containing 1 M Tris-HCl (pH 8.0), 0.5 M EDTA, 1 M NaCl, 10 μg/mL protease inhibitor and 1% Nonidet P-40 and the samples were separated by SDS-PAGE and were blotted onto a PVDF membrane, then blocked with 5% skim milk for 1 h. After blocking, the membranes were incubated with the primary antibody against METTL3 (1:1000) for 2 h, and HRP-conjugated anti-rabbit IgG (1:2000) for another 2 h, at 4 °C. Chemiluminescent detection was performed using e-BLOT system.

### 2.6. MeRIP-Seq and RNA-Seq Analysis

MeRIP-seq and RNA-seq were performed on METTL3 knockout (KO) and negative control (NC) HEK293T cells. Three independent biological replicates (separate culture wells of the same clonal cell line) were prepared for each condition.

Total RNA was extracted with TRIzol reagent (Invitrogen, Carlsbad, CA, USA, 15596018). The extracted total RNA was analyzed for concentration and integrity using the Qubit RNA BR assay (Thermo Fisher Scientific, Waltham, MA, USA, Q32852), and agarose gel electrophoresis. For MeRIP experiment, design the probe of rRNA, take about 10 ug total RNA, and after combining the probe with rRNA, remove the rRNA and DNA probe through the action of RNase H and DNase I at 37 °C. The purified RNA is processed with 10× RNA fragmentation buffer (Invitrogen, Carlsbad, CA, USA, AM8740).An amount of 1/20 of the RNA was reserved as input sample for RNA-seq, and the remaining 19/20 was subjected to immunoprecipitation (IP) sample for MeRIP-seq. The specific operation for MeRIP-seq is as follows: place the sample in a preheated thermal circulator and incubate it for 5 min at 70 °C. After purification, the fragmented RNA was subjected to m^6^A immunoprecipitation with 4 μg of m^6^A antibody (Synaptic Systems, Göttingen, Germany, 202003) which had been pre-incubated with 25 ul of protein A/G beads (Thermo Fisher Scientific, Waltham, MA, USA, 10002D and 10004D). The incubation was performed at 4 °C for 4 h with rotation. After immunoprecipitation, beads were collected and rinsed three times with chilled IP buffer (10 mM Tris-HCl pH 7.5, 150 mM NaCl, 0.1% NP-40), with each wash lasting 5 min at 4 °C under rotation. And m^6^A-containing fragments were eluted from the beads and recovered using TRIzol and a commercial cleanup kit (JIANSHI BIOTECH, Yuyao, Zhejiang, ChinaTR115-200). After purification, both m^6^A antibody-enriched RNA and partial of unenriched RNA after fragmentation, which was used as Input, was constructed library using VAHTS Universal V10 RNA-seq Library Prep Kit (Vazyme, Nanjing, Jiangsu, China) for Illumina according to the manufacturer’s protocol. The libraries were analyzed by an Agilent 2100 Bioanalyzer (Agilent Technologies, Santa Clara, CA, USA, G2946-60002) and quantified by real time PCR and then sequenced by the Illumina HiSeq PE150 platform (Illumina, San Diego, CA, USA).

The MeRIP sequencing services were provided by Shenzhen E-GENE Co., Ltd. Raw FASTQ files were evaluated for sequencing quality, duplication rates and adapter contamination via FastQC (v0.12.1), low-quality reads and adapters were trimmed with Trimmomatic (v0.38) and clean reads were aligned to the human genome (GRCh38/hg38, UCSC) with HISAT2 (v2.2.1). Methylation peak calling of each sample was identified separately using exomePeak (v2.16.0) (parameters: window width = 200 bp, estimated fragment length = 200 bp, sliding step = 30 bp, and minimum fold enrichment = 1.5). The differential peaks and the associated genes were authenticated using ChIPseeker (v1.30.2.4). The heatmap was generated in R (v4.1.1) using the pheatmap package. The genome-wide peak visualization was implemented through igvtools (v.2.10.0). KEGG and GO enrichment analysis was carried out via clusterProfiler (v4.4.2). Gene expression abundance was quantified as transcripts per million (TPM). The DESeq2 software was used to identify DEGs, with statistical significance defined as adjusted *p*-value < 0.05 and |log_2_ (fold change)| ≥ 0.585 (i.e., fold change ≥ 1.5) were identified as DEGs. All sequencing data are accessible via NCBI SRA under BioProject PRJNA1478046.

### 2.7. EBOV trVLPs and Luciferase Reporter Assays

Ebola virus-like particles (EBOV trVLPs) containing tetracistronic minigenomes was employed to simulate the life cycle of EBOV in a biosafety level 2 (BSL-2) laboratory [36]. Briefly, HEK293T cells, growing in a 6-well plate, were co-transfected with the indicated amount plasmids for P0: pCAGGS-NP (125 ng), pCAGGS-VP35 (125 ng), pCAGGS-VP30 (75 ng), pCAGGS-L (1000 ng), pCAGGS-T7 (250 ng), p4cis-vRNA-RLuc(250 ng) and 25 ng of the PGL3-promoter plasmid which carries a firefly luciferase reporter gene serving as an internal control for transfection efficiency. P0 cells were transfected for 48 h and the cell lysate and supernatant were collected.

For P1, cells were transfected with the plasmids pCAGGS-NP (125 ng), pCAGGS-VP35 (125 ng), pCAGGS-VP30 (75 ng), pCAGGS-Tim1 (250 ng), pCAGGS-L (1000 ng), and 25 ng of the PGL3-promoter plasmid for 24 h, whereafter, the cells were infected with the supernatant collected from p0 cells. P1 cells were harvested after 48 h of infection.

Lipofectamine 3000 was used as transfection reagent, and luciferase activity was assessed by using the Dual-Luciferase^®^ Dual Luciferase Report Gene Assay System according to the manufacturer’s protocol. Simultaneously, P0/P1 cells were lysed with 250 μL of 1× Passive Lysis Buffer (PLB) on a shaker (180 rpm, 15 min). Then, 20 μL of cell lysate and 100 μL of Luciferase Assay Reagent II were mixed in the centrifuge tube, and firefly luminescence (R1) was recorded. Then renilla luminescence (R2) was measured after 100 μL of Stop & Glo^®^ Reagent was added. The R2/R1 ratio was calculated to normalize for transfection efficiency and to control for any variation in cell number or viability between samples, and used to assess viral replication.

### 2.8. Statistical Analysis

Each experiment was performed at least in triplicate, and statistical computations were conducted with GraphPad Prism 8. Data are presented as mean values with SDs. Intergroup comparisons between two conditions were assessed using a two-tailed unpaired Student’s *t*-test, with statistical significance defined as *p* < 0.05.

## 3. Results

### 3.1. Summarization of m^6^A Methylation Pattern Alterations Due to METTL3 Silencing

**Figure 1:**
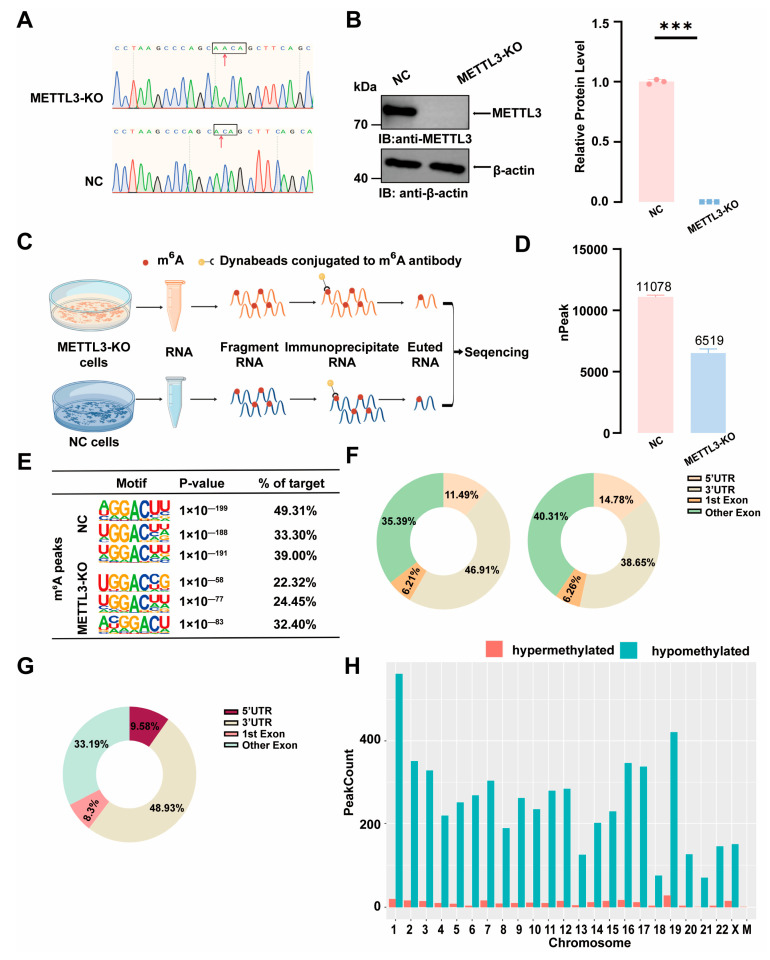
Summarization of m^6^A methylation pattern alterations due to METTL3 deficiency. (**A**) Sequencing analysis of the target gene sequence in METTL3 knockout cells. (**B**) METTL3 expression was evaluated via immunoblotting with β-actin serving as the control and its gray scale maps. (**C**) Experimental design for the m^6^A methylation modification analysis in this study. (**D**) The number of m^6^A peaks acquired in NC or METTL3-KO cells. (**E**) The top three m^6^A methylation motif sequences in NC or METTL3 KO cells. (**F**) Pie charts showing m^6^A peaks distributed in gene contexts of NC or METTL3 KO cells. (**G**) The proportion of discrepant m^6^A peaks in the total peak of the gene elements. (**H**) Hypermethylation or hypomethylation m^6^A peaks on chromosomes were shown. *** *p* < 0.001.

To study the global mRNA m^6^A methylation alteration pattern driven by METTL3 silencing, a METTL3 knockout (METTL3-KO) HEK293T cell line was generated via CRISPR-Cas9 gene-editing technology. As shown in Figure 1A,B, a one base pair insertion which caused a frameshift mutation and resulted in the ablation of METTL3 protein was confirmed, as verified by sanger sequencing and immunoblotting. Then, the METTL3-KO cell line or negative control (NC) cell line were subjected to MeRIP-seq analysis. Figure 1C showed the experimental workflow. Based on the sequencing data, an average of 6552 and 11,057 m^6^A modification peaks were identified in the METTL3-KO and NC cell line samples (Figure 1D), respectively, indicating that loss of METTL3 leads to a reduction of detectable global m^6^A modification, accompanied by an alteration of major m^6^A-modified motif characteristics (Figure 1E). We then analyzed the distribution pattern of m^6^A peaks in these two samples and found that METTL3 abstention led to a decrease m^6^A peaks at the 3′UTR region (38.65% vs. 46.91%), and an increase at the other exons region (40.31% vs. 35.7%) and 5′UTR region (14.78% vs. 11.49%) (Figure 1F). Since METTL3-KO cells exhibited different peaks, we sought to analyze the distributed pattern of different peaks and found that they were primarily located at the 3′UTR region (48.93%) and other exons region (33.19%) (Figure 1G). And as shown in Figure 1H, different peaks were detected on all chromosomes, and were particularly abundant on chromosomes 1, 19, 2, 3, 16 and 17. These data collectedly exhibited an overview of METTL3-mediated alterations in mRNA m^6^A methylation pattern, suggesting that METTL3 deletion significantly decreased the m^6^A modification in mRNAs and altered the modified sequences preference in cells.

### 3.2. Identification of METTL3-Mediated Differentially Methylated Genes (DMGs) and Functional Enrichment Analysis

**Figure 2:**
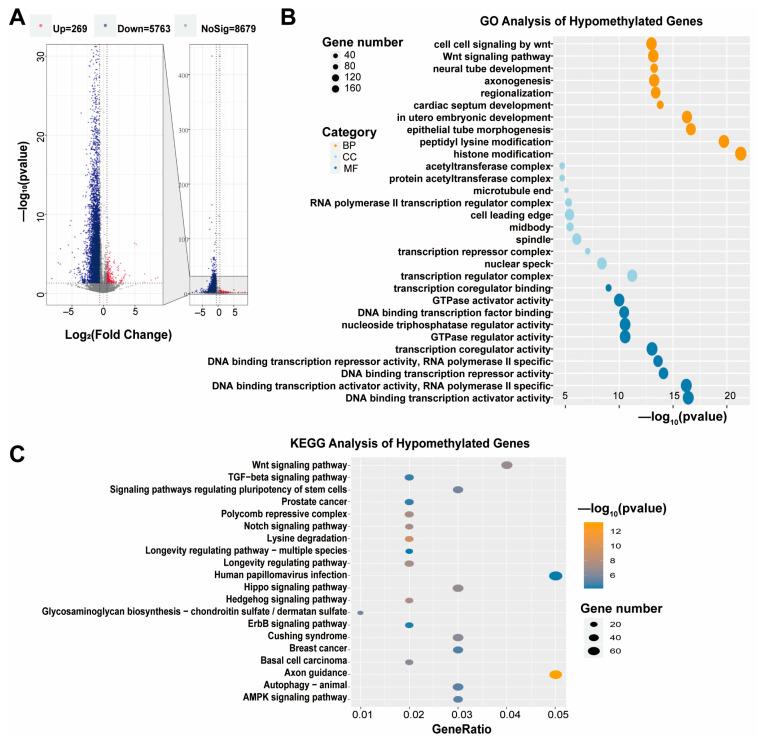
Identification and functional enrichment analyses of METTL3-mediated DMGs. (**A**) Volcano plot showing of the DMGs. (**B**,**C**) KEGG pathway and GO pathway enrichment analyses of the hypomethylated genes. The GO category includes biological process (BP), cell component (CC), and molecular function (MF). Significantly enriched categories were ranked by *p* value, and the top ten of GO terms and top twenty terms of KEGG were shown. The enriched genes number is represented for the size of dots. The larger the dot was, the more genes were enriched.

The different m^6^A-modified mRNAs caused by METTL3 deletion then were annotated to identify genes. As shown in Figure 2A, 5763 hypomethylated genes and 269 hypermethylated genes were identified after METTL3 silencing. The 5763 hypomethylated genes caused our interest, as METTL3 is a methyltransferase, and to determine the biological processes and pathways these genes involved, GO analysis and KEGG pathway enrichment analysis were performed. The top ten of GO terms in the BP, MF, and CC categories and top twenty terms of KEGG were shown (Figure 2B,C). The results indicated that the BP terms were primarily enriched in histone modification, peptidyl–lysine modification, and the Wnt signaling pathway. CC terms were mainly observed enriched in transcription regulator complexes, nuclear specks, and transcription repressor complexes. MF terms were mainly enriched in DNA-binding transcription activator activity, RNA polymerase II specific, and DNA-binding transcription repressor activity. For KEGG pathway enrichment, the results showed that the genes were primarily enriched in axon guidance, human papillomavirus infection, and the AMPK signaling pathway, etc.

### 3.3. Identification of the DEGs After METTL3 Silencing

**Figure 3:**
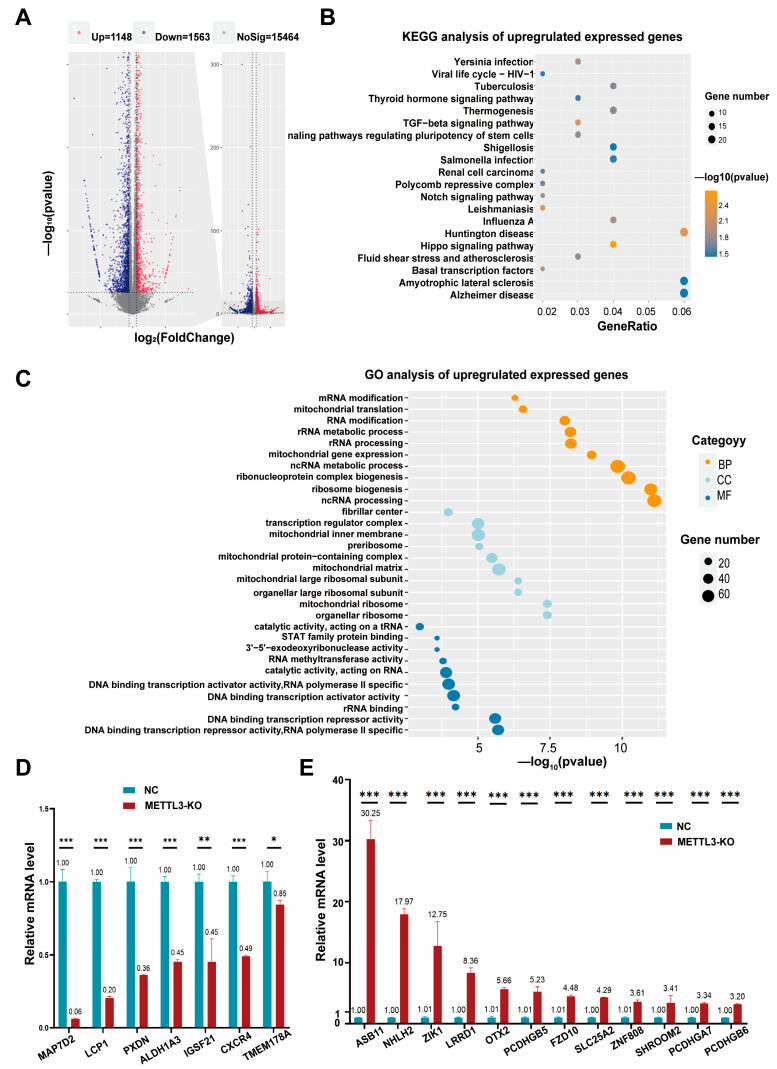
Identification of DEGs and functional enrichment analyses. (**A**) Volcano plot depicting DEGs between METTL3-KO and NC cells. (**B**,**C**) GO and KEGG pathway enrichment analyses of the upregulated expressed genes. (**D**,**E**) qRT-PCR validation of the upregulated (**D**) or downregulated (**E**) genes. All dates are presented as mean ± SD (*n* = 3 biological replicates). * *p* < 0.05, ** *p* < 0.01, *** *p* < 0.001, Student’s *t*-test.

Considering that m^6^A modification affects the fate of mRNA by altering its stability, translation efficiency and pre-mRNA splicing [27,37,38], to reveal the DEGs after METTL3 silencing, RNA sequencing was performed. According to the data, a total of 2711 genes expression were significantly altered, with 1148 upregulated and 1563 downregulated (Figure 3A). To further explore the potential function of these genes, the GO and pathway annotation analyses were performed, and here were shown the enrichment result of upregulated genes. They were primarily enriched in ribosome biogenesis, mitochondrial gene expression, ncRNA metabolic processes, and ribonucleoprotein complex biogenesis in BP terms. CC enrichment analysis showed that mitochondrial ribosomes, organellar ribosomes, and transcription regulator complexes were the primary terms, and the main MF terms were RNA methyltransferase activity, rRNA-binding, and DNA-binding transcription activator/repressor activity, etc. (Figure 3B). According to the KEGG pathway analysis, *Yersinia* infection, tuberculosis, *Shigellosis*, salmonella infection, influenza A, the HIV-1 life cycle and leishmaniasis were significantly enriched (Figure 3C), indicating that METTL3-mediated DEGs might participate in defense response against bacterial and viral pathogens. Furthermore, a subset of upregulated or downregulated genes is validated by using qRT-PCR (Figure 3D,E).

### 3.4. Conjoint Analysis of DMGs and DEGs Identified METTL3 Was Involved in Immune Response Against Infection

**Figure 4:**
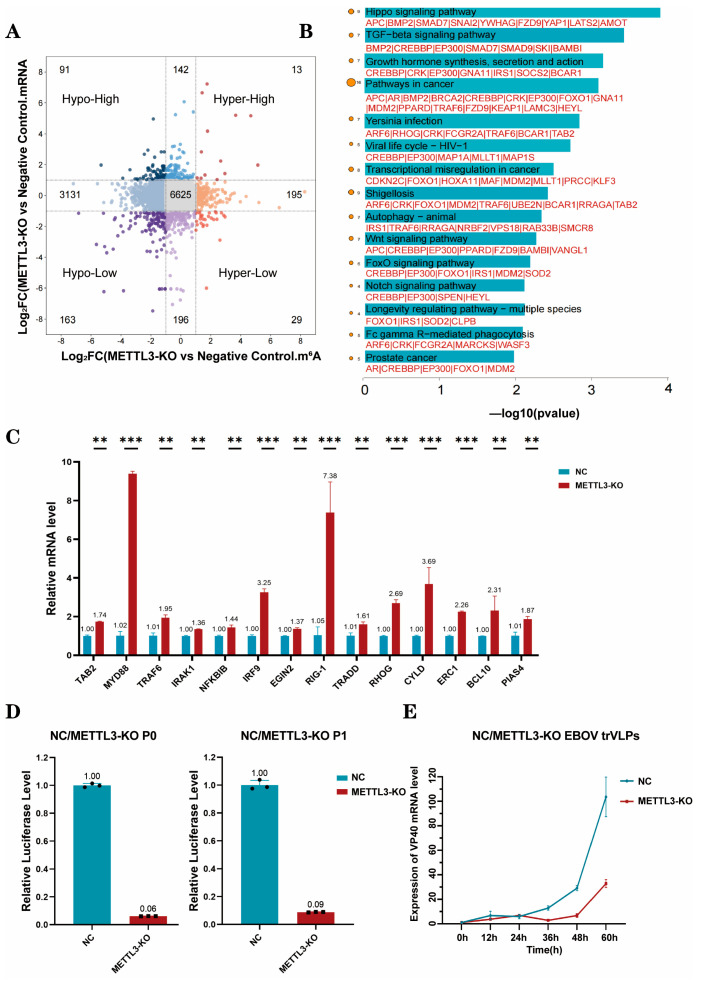
METTL3 are involved in immune response against infection. (**A**) A conjoint analysis of DEGs and DMGs, showing of 13 hypermethylated-upregulated, 91 hypomethylated-upregulated, 163 hypomethylated-downregulated and 29 hypermethylated-downregulated genes. (**B**) KEGG pathway analysis of the identified 91 hypomethylated-upregulated genes, and the key genes were shown under the pathway terms. The size of bubbles represents the number of genes enriched in this KEGG pathway. (**C**) qRT-PCR validation of the immune-related pathways core genes. (**D**) Luciferase activity in tetracistronic minigenome-transfected METTL3-KO versus NC cells (P0 or P1, 72 h post-transfection) and the cells were lysed and the luciferase activity can represent the replication level of trVLPs. (**E**) METTL3 KO or NC cells were transfected with trVLPs, after which the viral VP40 mRNA level is determined by qRT-PCR at the indicated time points. Data were from three independent experiments, which presented as mean ± SD. ** *p* < 0.01, *** *p* < 0.001.

Because m^6^A modification influences mRNA stability, translation or splicing, and causes a crucial impact on gene expression, the DMGs and DEGs were subjected to integrated analysis and were classified into four groups, including 13 hypermethylated-upregulated genes, 91 hypomethylated-upregulated genes, 163 hypomethylated-downregulated genes and 29 hypermethylated-downregulated genes, as shown in Figure 4A. KEGG enrichment analysis of all four gene categories were performed. Given that m^6^A modification can modulate mRNA stability in a reader-dependent manner—either promoting degradation via YTHDF2 or enhancing stability via IGF2BPs [38,39,40]—here we employed hypo-upregulated genes for further analysis to investigate the biological function in which they were involved. As a result, these hypo-upregulated genes were primary involved in pathogen infection and immune-related pathways, such as *Yersinia* infection, *Shigellosis* and the HIV-1 viral life cycle, which were consistent with the enrichment of upregulated genes aforementioned, and here the related genes were shown under the pathway terms (Figure 4B). Among these genes, several core genes, such as myeloid differentiation primary response 88 (*MYD88*), retinoic acid-inducible gene 1 (*RIG-1*), **cylindromatosis; ubiquitin carboxyl-terminal hydrolase** (*CYLD*) and interferon regulatory factor 9 (*IRF9*), were selected for qRT-PCR verification (Figure 4C). These data indicated that METTL3 manipulated these genes which might inhibited innate immune response. To test this supposition, Ebola virus trVLPs (EBOV trVLPs) carrying a luciferase reporter, which can model EBOV lifecycle, evaluate EBOV infection and replication, was employed here to investigate the role of METTL3 in EBOV infection [36]. As indicated in Figure 4D, E, METTL3 KO cells or NC cells were subjected to EBOV trVLPs infection, and the results showed that METTL3 KO cells represented a lower luciferase level or viral VP40 mRNA level, suggesting that METTL3 KO cells suffered less from EBOV trVLPs infection.

## 4. Discussion

Among eukaryotic RNA modifications, m^6^A methylation is the most conservative and abundant modification [38,41], which has been established as an essential regulator of transcription initiation, mRNA splicing, stability and mRNA transportation, causes mRNA expression alteration [39,41,42,43]. METTL3 is the major writer of m^6^A modification and has been extensively studied in many [43] reports [42,43]. Indeed, it is well established that METTL3 contributes critically to embryonic development [16,17,18,19], cancer progress [1,2,13,14,25,26] or even antiviral immune response [44,45,46], by manipulating characteristic genes expression. Therefore, delineating the mRNA methylation and gene expression profile altered by METTL3, may help to illuminate the initiation and development of related diseases and offer avenues for clinical intervention. Nevertheless, to the best of our knowledge, a comprehensive view of both the m^6^A landscape and the corresponding transcriptome alterations governed by METTL3 has remained unavailable.

In this study, we characterized, in mRNA methylation, pattern changes; gene expression alteration, in the case of METTL3 deletion, was revealed. By employing MeRIP-Seq and RNA-Seq analyses, a total of 6032 DMGs (5763 hypomethylated/269 hypermethylated) and 2711 DEGs (1148 upregulated/1563 downregulated) upon METTL3 ablation were identified (Figure 2A and Figure 3A). And many methylation alteration genes were enriched in the Wnt signaling pathway, regulating pluripotency of stem cells, prostate cancers and breast cancers (Figure 2C), which align with the established roles of METTL3 in embryogenesis and tumorigenesis, supported the credibility of our sequencing data as well. Additionally, the KEGG analysis showed that many important genes, also mainly involved in pathogen response pathways, including *Yersinia*, tuberculosis, salmonella, *Shigellosis* infection, viral life cycle-HIV-1 and influenza A (Figure 3C and Figure 4B), caused our great interest, so the core genes such as *MYD88*, *RIG-1*, *CYLD* and *IRF9* were selected for further verification. Among the genes upregulated upon METTL3 knockout, *MYD88* exhibited a particularly notable increase in expression. As a central adaptor protein in the TLR signaling cascade, elevated *MYD88* expression promotes NF-κB pathway activation; its elevated expression is expected to potentiate NF-κB activation and promote the release of pro-inflammatory mediators [47]. This process may contribute to strengthening the host’s innate immune defense against pathogens. Furthermore, *RIG-1* (encoded by *DDX58*) was also substantially upregulated. *RIG-1* functions as a cytoplasmic sensor for viral RNAs [32], recognizing RNA molecules that carry a 5′-triphosphate moiety. Upon detecting viral invasion, *RIG-1* triggers downstream signaling cascades that initiate innate immune responses [48,49,50], inducing the production of type I interferons and various inflammatory cytokines, thereby assisting the host in combating viral infections [50]. And EBOV infection and proliferation assays were performed, ultimately determined that METTL3 deletion resulted in significantly attenuated EBOV proliferation and infection capacity (Figure 4D,E), indicating that METTL3 knockout enhanced host innate antiviral immune response. Further mechanistic experiments are required to establish direct causality.

We focused on genes that were hypomethylated yet upregulated upon METTL3 knockout. KEGG enrichment analysis revealed accidentally that the hypo-upregulated genes were significantly enriched in innate immune-related pathways such as *Yersinia* infection, *Shigellosis*, HIV-1 viral life cycle, whereas the other three groups include hyper-upregulated, hypo-downregulated, and hyper-downregulated, showing no such enrichment. This unexpected finding suggests that hypo-upregulated genes may be associated with innate immune responses upon METTL3 depletion, and thus we focused on this gene set for subsequent analysis. Although m^6^A modification generally promotes mRNA stability, hypomethylation would be expected to lead to downregulation; however, these genes showed the opposite trend, suggesting that METTL3 deficiency may regulate their expression through non-canonical mechanisms such as compensatory transcriptional activation. This pattern differs from the general expectation that hypomethylation leads to downregulation. Similar observations have been reported previously: Wang et al. (2019) found that upon loss of DNA methylation, cells can activate specific genes through epigenetic compensation mechanisms such as H3K27me3 redistribution [51]. Additionally, enhancer hypomethylation of immune response genes has been shown to sustain transcriptional activation in intestinal stem cells [52]. Our data suggest that the immune-related enrichment among hypomethylated–upregulated genes upon METTL3 depletion may involve comparable compensatory regulatory mechanisms. The present study was mainly conducted in HEK293T cells, a widely accepted model in the field. The METTL3 knockout cell line used in this study was derived from a single positive clone (clone #10). During screening, multiple single clones were isolated by limited dilution, but only this one exhibited complete loss of METTL3 protein. Stable METTL3 knockout cell lines are difficult to obtain in most cell lines, consistent with the essential role of METTL3 in cell proliferation and organ development [35,53,54]. Consequently, we were unable to obtain a second independent clone for parallel validation. We admit that a single clone may be subject to potential effects from lentiviral integration site or sgRNA off-target activity, which represents a limitation of this study. Nevertheless, we recognize the necessity of validating our major findings in more physiologically relevant immune cells. To this end, we attempted to establish METTL3 knockout in THP-1 (human monocytic), Huh7, and HepG2 (human hepatoma) cell lines, but without success. Specifically, METTL3 knockout was lethal in Huh7 and HepG2 cells (no viable clones obtained), indicating an irreplaceable role of METTL3 in cell survival in these lines. THP-1 cells exhibited extremely low transduction efficiency (<5%) with the lentiviral CRISPR system, possibly due to low expression of viral receptors or high expression of exogenous DNA degradation enzymes. We also attempted MeRIP-qPCR validation for key genes including *MYD88*, *RIG-1*, *CYLD*, and *IRF9*, but failed to obtain reliable signals, likely because of low m^6^A abundance on these transcripts or insufficient antibody pull-down efficiency. Future approaches may include using high-affinity antibodies, increasing RNA input, or adopting Nanopore direct RNA sequencing. The critical functions of METTL3-mediated m^6^A modification in the regulation of gene expression have been corroborated by multiple independent studies. For example, one study reported that METTL3 deletion in mouse embryonic stem cells gives rise to widespread upregulation of transposable elements, indicated its significant role in early embryonic development and cell fate determination [55]. In another study, it was shown that silence of METTL3 in AGS cells altered the expression profile of many effector molecules that served key roles in cell proliferation, suggesting METTL3 played a potential role in gastric cancer progression [56]. More importantly, there were reports which showed that METTL3-mediated m^6^A modification secured antiviral immunity by enhancing mRNA stability or protein translation [29,30,31,44]. Our finding supported these studies to a certain extent. Distinct from previous MeRIP-seq studies of METTL3 in cancer cells [26,56], stem cells growth [35], or liver tissues [51,53], our work specifically links METTL3 depletion to altered expression of innate immune-related genes, with hypo-upregulated genes significantly enriched in pathogen infection and immune pathways. But it is important to indicate that, such as gene deletion strategy, culture condition and cell heterogeneity can lead to the variety of data among labs. Here, we addressed that the methylation alteration pattern and gene expression profile altered by METTL3 increased the overall understanding of METTL3-mediated m^6^A modification in disease pathways, highlighted the potential of METTL3 and the exposed genes as therapeutic targets for combating pathogens.

## Data Availability

The datasets analyzed in this study are publicly available in the SRA under accession numbers PRJNA1478046. All other data generated or analyzed are included in the article and are available from the corresponding author upon request.

## Appendix A. Primer Sequence

**Primer**	**Sequence**
ALDH1A3-F	CTGCTACAACGCCCTCTATGCA
ALDH1A3-R	GTCGCCAAGTTTGATGGTGACAG
ASB11-F	GGCAAAAGTGCGCTTGATCTGG
BCL10-F	AGCGCGACCATCGGAGAGGT
BCL10-R	ACATGCTCGACCGAGACACTTC
BCL10-R	TGTGGCCGCAGAATGGCAGG
CXCR4-F	CTCCTCTTTGTCATCACGCTTCC
CXCR4-R	GGATGAGGACACTGCTGTAGAG
CYLD-F	AATCCGCTCTTCCCAGTAG
CYLD-R	GGAGGCCATATACCCTGTCAGGTC
EGIN2-F	AACATCGAGCCACTCTTTGAC
EGIN2-R	TCCTTGGCATCAAAATACCAG
ERC1-F	GGAATCAGCCAGGACCAATGCT
ERC1-R	CCTTTTCTGCCAGAGACTGCTG
FZD10-F	GAACACGGACAAGCTGGAGAAG
FZD10-R	GGCGTTCGTAAAAGTAGCAGGC
GAPDH-F	AAGGTCATCCCTGAGCTGAAC
GAPDH-R	ACGCCTGCTTCACCACCTTCT
IGSF21-F	AGAACTACCGCAAGCGAGAGGA
IGSF21-R	CTGATGCCAGGACCACCTTCTC
IRAK1-F	GGACACGGACACCTTCAGC
IRAK1-R	CAGCCTCCTCTTCCACCAG
IRF9-F	GCCTTTGCCCCATCCCCATCTC
IRF9-R	CCCCTGGCCCTGGAAGTACTGG
LCP1-F	GCTCTTGCTGAGGTGGGCTAAT
LCP1-R	TCTCCTTTTGGAGCCACCTGCT
LRRD1-F	TTGAGAAATTTGGTTAGTTTACATGC
LRRD1-R	GCAGATAGCACTAGGTAGAGCTG
MAP7D2-F	CTGGAGGCATTCCTAAGAGACC
MAP7D2-R	GTAAGCGGTACTTCACAGGAGAC
MYD88-F	GAGGCTGAGAAGCCTTTACAGG
MYD88-R	GCAGATGAAGGCATCGAAACGC
NFKBIB-F	GCTCATGCTGATGAATCACAGC
NFKBIB-R	GATGTTACGCTTGTTCTGTGG
NHLH2-F	ATCCGCGTGGAAGCCTTCAACT
NHLH2-R	GTCCAGGACGTGGTTGAGATAG
NIBAN1-F	CCACCAAAGACAGTGTCCAGCT
NIBAN1-R	AAGCGGCTCTTGAGATCCTGCA
OTX2-F	GGAAGCACTGTTTGCCAAGACC
OTX2-R	CTGTTGTTGGCGGCACTTAGCT
PCDHGB5-F	CAACACGGACTGGCGTTTCTCT
PCDHGB5-R	GATCATGGCTTGCAGCATCTCTG
PIAS4-F	CTTTAATATGCTGGATGAGCTG
PIAS4-R	CTCCTTGACCAGTGCCTTGCAC
PXDN-F	CGGACATTGCAGCTCATTCAGG
PXDN-R	CCGACAGGTTTGCGATGAGGTT
RHOG-F	CCGCTCTCACTTCCTTCTC
RHOG-R	ACCACCACGCACTTGATG
RIG-1-F	GACTGGACGTGGCAAAACAA
RIG-1-R	TTGAATGCATCCAATATACACTTCTG
SLC25A2-F	CTGCTTCCTGAAGACATACGCC
SLC25A2-R	CACTTTCCTGACAAACTGCTGGC
TAB2-F	GACCTGCCCTGGAAAAGAAGT
TAB2-R	CTCTTCTGTCCAAGCATTTTCTGG
TMEM178A-F	CCATCCGCTTGCGAAACATTCC
TMEM178A-R	ACAATGCAGCCGCAGAGAAGGA
TRADD-F	CGCTCTGTGGGTCTCAAATGGC
TRADD-R	AGTCCTCTGCCAGGCTGGTGAG
TRAF6-F	AACTGTGCTGTGTCCATGGC
TRAF6-R	CAGTCTCATGTGCAACTGGG
VP40-F	GGAGGCCATATACCCTGTCAGGTC
VP40-R	GCCTGGTGTGTGGCTGGCAT
ZIK1-F	CTTGATGAGGCTCAGAGACTCC
ZIK1-R	CCTGCCTTTGACTGTGACACTC
ZNF808-F	GGAAGTGATGTTGGAGAACTACAG
ZNF808-R	GCAATGTCCCTGTGTGGATCAC
